# The causal role of multiple negative emotions in chronic respiratory diseases: A two-sample Mendelian randomized study

**DOI:** 10.1016/j.clinsp.2025.100819

**Published:** 2025-11-07

**Authors:** Hui Chen, Jinping Zhu, Zewen Yang, Yimin Zha, Junjie Yang

**Affiliations:** Department of Respiratory and Critical Care Medicine, Shenzhen Bao’an District Songgang People’s Hospital, Shenzhen, People’s Republic of China

**Keywords:** Chronic respiratory diseases, Mendelian randomization, Negative emotions

## Abstract

**Background:**

The relationship between negative emotions and chronic respiratory diseases remains largely unexplored. In this study, we employ Mendelian Randomization (MR) to investigate the potential causal relationship between negative emotions and chronic respiratory diseases.

**Methods:**

The data used in this study were derived from the publicly available GWAS summary statistics in the IEU Open GWAS database, including 12 negative emotions and 6 chronic respiratory diseases: asthma, Chronic Obstructive Pulmonary Disease (COPD), bronchiectasis, sarcoidosis, sleep apnea syndrome, and Idiopathic Pulmonary Fibrosis (IPF). Five models were established for MR analysis. Leave-one-out sensitivity analysis, MR pleiotropy residual sum, and MR-PRESSO, and Cochran's *Q* test were utilized to confirm heterogeneity and pleiotropy.

**Results:**

This study indicates a causal relationship between negative emotions and chronic respiratory disease. Specifically, neuroticism, experiencing mood swings, feeling nervous and feeling worried were associated with an increased risk of asthma. Additionally, neuroticism, feeling miserable, experiencing mood swings, feeling guilty, and worrying too long after an embarrassing experience were associated with an increased risk of COPD. Similarly, neuroticism, experiencing mood swings, and feeling hurt increased the risk of sleep apnea syndrome. No causal association was observed between negative emotions and bronchiectasis, IPF, or sarcoidosis. In MVMR analysis, negative emotions were also associated with asthma and sleep apnea syndrome, while only the causal effect of experiencing mood swings persisted with COPD.

**Conclusion:**

This study indicated a causal effect between negative emotions and chronic respiratory diseases, highlighting the importance of early intervention measures targeting negative emotions in the prevention of chronic respiratory diseases.

## Introduction

Emotions are continuously elicited by stimuli from both internal and external environments, shaping individuals' attitudes, experiences, and corresponding behavioral responses to objective circumstances, significantly impacting health outcomes.^1^ Amidst the COVID-19 pandemic, heightened life stressors, and lifestyle changes, the prevalence of negative emotions has noticeably escalated^2^ persistent difficulties in regulating negative emotions may predispose individuals to develop mental disorders, thereby imposing substantial socioeconomic burdens. Recent scholarly interest has centered on exploring the role of emotions and psychological factors in disease etiology. Empirical evidence has established a robust association between negative emotions and the onset and progression of various ailments, including female reproductive disorders, psoriasis, and gastroesophageal reflux.^3^ Mechanistically, these associations are predominantly categorized into behavioral and molecular dimensions. Behaviorally, individuals experiencing negative emotions often exhibit unfavorable lifestyle choices such as increased smoking, alcohol consumption, poor dietary habits, and reduced physical activity, all contributing to disease susceptibility. At the molecular level, prolonged negative emotions disrupt the homeostasis of the autonomic nervous system, resulting in dysregulated hormonal and immune functions that foster inflammatory processes and subsequent disease pathogenesis.^4^ Interventions targeting negative emotions in early stages have been shown to mitigate the incidence and mortality of associated diseases.

Chronic respiratory diseases encompass a spectrum of conditions affecting airway function, notably including asthma, bronchiectasis, Chronic Obstructive Pulmonary Disease (COPD), Idiopathic Pulmonary Fibrosis (IPF), sarcoidosis, and sleep apnea syndrome. These diseases are characterized by structural and functional alterations in lung tissue attributed to dysregulated inflammatory responses. A delicate interplay exists between the intricate functions of the human brain and the respiratory system, where emotions influence disease development and progression through the “lung-brain axis”.^5^ Extensive research has explored the complex interrelationships between respiratory diseases and various emotional and psychological factors.^6^ Observational studies have consistently underscored the significant impact of psychological disorders on lung function and their pivotal role in disease progression. Severe depressive symptoms correlate with accelerated declines in lung function and increased mortality rates from respiratory disorders.^7^ Notably, severe depression exacerbates Th2 and Th17 cell cytokine production in allergic rhinitis and asthma patients, with Interleukin (IL)-5 and IL-17 levels directly correlating with the severity of depressive and anxiety symptoms.^8^ Individuals with COPD experiencing depression and anxiety exhibit worsened respiratory symptoms and exacerbations, significantly compromising health-related quality of life.^9^ Despite compelling evidence indicating a link between psychological disorders and chronic lung diseases, research specifically investigating the association between negative emotions and chronic respiratory conditions remains sparse. Furthermore, inherent limitations of observational studies, including potential residual confounding and reverse causality, may obscure the understanding of causal relationships.^10^ Therefore, investigating the causal relationship between negative emotions and chronic respiratory diseases is of significant clinical importance.

Mendelian Randomization (MR) analysis stands as a widely utilized methodological approach to elucidate potential causal relationships between exposures and outcomes.^11^ By leveraging genetic variants as Instrumental Variables (IVs), MR analysis helps mitigate confounding factors and unravel reverse causality biases.^12^ Therefore, we aimed to assess the causal relationship between negative emotions and chronic respiratory diseases through MR analysis, providing a scientific basis for the prevention of these diseases.

## Methods

### Study design

**Fig. 1:**
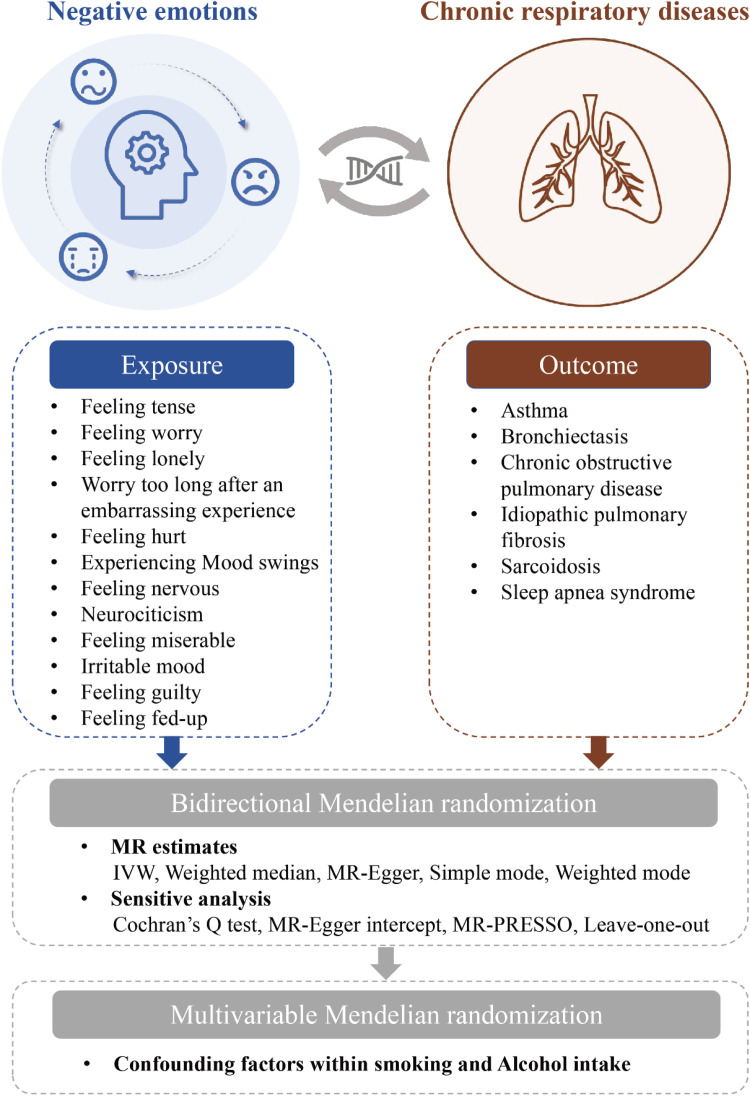
A flowchart illustrating the study design process. IVW, Inverse Variance-Weighted; MR, Mendelian Randomization; MR-PRESSO, Mendelian Randomization Pleiotropy Residual Sum and outlier.

The present study employed two-sample MR and Multivariable MR (MVMR), adhering to the STROBE-MR guidelines. The specific workflow is illustrated in Fig. 1. Initially, we utilized two-sample MR to investigate bidirectional associations between 12 negative emotions (feeling tense, feeling worry, feeling lonely, worry too long after an embarrassing experience, feeling hurt, experiencing mood swings, feeling nervous, neurociticism, feeling miserable, irritable mood, feeling guilty, feeling fed-up) and six common chronic respiratory diseases (asthma, bronchiectasis, COPD, IPF, sarcoidosis, sleep apnea syndrome). Reverse MR analyses were conducted for positively associated negative emotions identified in two-sample MR to assess the reverse causal relationships. For exposures with significant causal relationships, MVMR analysis was performed using the MVMR-Inverse Variance Weighted (IVW) method. We considered smoking and alcohol intake as potential confounding factors in the exposure-outcome relationship and adjusted for these confounders using three different models. A p-value below 0.05 was considered statistically significant.

### Data sources

In this study, Genome-Wide Association Studies (GWAS) summary data were curated from the IEU Open GWAS database, providing statistical insights into six common chronic respiratory diseases. Summary statistics for 12 negative emotions were obtained from Nagel et al., available in the Open-GWAS database.^13^ The Eysenck Personality Questionnaire Revised Short Form (EPQ-RS) was used to measure the emotions. The responses to each item were summed, weighted, and averaged, followed by standardization of the total score. Table 1 provides comprehensive details regarding GWAS summary data, encompassing the core exposure and outcome variables for the MR investigation. These datasets were sourced from the IEU Open GWAS project (https://gwas.mrcieu.ac.uk), with each cohort rigorously ethically approved and obtaining explicit informed consent from all participants.

**Table 1:** Overview of the GWAS datasets used in the MR analysis.

Contribution	Traits	Sample size	Number of SNPs	Ancestry	GWAS ID
Exposure	Feeling tense	371.318	10.824.652	European	ebi-a-GCST006952
	Feeling worry	372.869	10.824.850	European	ebi-a-GCST006950
	Feeling lonely	376.352	10.824.519	European	ebi-a-GCST006942
	Worry too long after an embarrassing experience	367.725	10.824.870	European	ebi-a-GCST006946
	Feeling hurt	372.047	10.824.675	European	ebi-a-GCST006951
	Experiencing Mood swings	373.733	10.824.841	European	ebi-a-GCST006944
	Feeling nervous	373.121	10.824.842	European	ebi-a-GCST006948
	Neurociticism	380.506	10.824.976	European	ebi-a-GCST006940
	Feeling miserable	376.097	10.824.771	European	ebi-a-GCST006943
	Irritable mood	366.726	10.824.680	European	ebi-a-GCST006941
	Feeling guilty	373.380	10.824.730	European	ebi-a-GCST006945
	Feeling fed-up	374.971	10.824.792	European	ebi-a-GCST006947
Outcome	Asthma	218.792	16.380.466	European	finn-b-ASTHMA_HOSPITAL_MAIN
	Bronchiectasis	187.830	16.380.375	European	finn-b-J10_BRONCHIECTASIS
	COPD	218.792	16.380.466	European	finn-b-COPD_HOSPITAL
	Idiopathic pulmonary fibrosis	198.014	16.380.413	European	finn-b-IPF
	Sarcoidosis	217.758	16.380.463	European	finn-b-D3_SARCOIDOSIS
	Sleep apnea syndrome	217.955	16.380.465	European	finn-b-G6_SLEEPAPNO
Confounders	Current tobacco smoking	462.434	9.851.867	European	ukb-b-223
	Alcohol intake	462.346	9.851.867	European	ukb-b-5779

SNPs, Single Nucleotide Polymorphism.

### Selection of IVs

MR analysis utilizes IVs to rigorously examine causal relationships between exposures and outcomes. The selection of Single Nucleotide Polymorphisms (SNPs) as exposure IVs necessitates strict adherence to three fundamental assumptions^14^^,^^15^ 1) The chosen SNP must exhibit a strong and unequivocal correlation with the exposure, as an effective tool for assessing causal relationships. 2) The selected SNP must be unrelated to any potential confounding variables that could distort the true association between exposure and outcome. 3) The SNP used as an instrument should influence the outcome solely through its effect on the exposure. These stringent prerequisites collectively underpin the reliability and validity of MR analysis.

Following these principles, we initially screened SNPs associated with negative emotions at a genome-wide significance threshold (*p* < 5 × 10^–8^) for MR analysis. To ensure independence among the SNPs, Linkage Disequilibrium (LD) clumping was performed using the 1000 Genomes Project reference panel (R² < 0.001 within 10,000 kb distance).^16^ Additionally, we carefully excluded palindromic SNPs, SNPs significantly associated with outcome variables (*p* < 0.05), and SNPs not present in the GWAS dataset for the outcome variable. Finally, to mitigate bias from weak instruments, we retained SNPs with an F-statistic > 10 for subsequent analyses.^17^

### Statistical methods

For SVMR, we first identified outliers using MR-PRESSO and subsequently removed these outliers. We then employed the IVW random-effects method to assess the causal impact of negative emotions on chronic respiratory diseases. Given that IVW requires all IVs to be valid for unbiased estimation, we also conducted MR analyses using four alternative methods ‒ Weighted Median, MR-Egger, Simple mode, Weighted mode ‒ to evaluate the robustness of the present findings. In MVMR, we extended the IVW-MR method by employing Multivariate Weighted Linear Regression (variable independent and random-effects model), setting the intercept term to 0.

A series of sensitivity analyses was conducted. Initially, we assessed heterogeneity using Cochran's *Q* test. In cases of significant heterogeneity among IVs (Q_pval well below 0.05), we estimated the MR effect size using a random-effects model; otherwise, a fixed-effects model was used. Subsequently, we employed MR-Egger intercept and MRPRESSO tests to detect horizontal pleiotropy. The False Discovery Rate (FDR)-corrected p was calculated in each set of results, and adjusted *p* < 0.05 was considered statistically significant. The causal effect is considered significant if the weighted median and MR-Egger results are in agreement with the IVW results. Finally, leave-one-out sensitivity analyses were conducted to assess result robustness.

The MR analyses were carried out using TwoSampleMR (version 0.5.7) and MRPRESSO (version 1.0) in R (version 4.3.2). Results are presented as Odds Ratios (ORs) with 95 % Confidence Intervals (95 % CIs). *R* version 4.3.2 was used to perform all statistical calculations.

## Results

### Selection of IVs associated with negative emotions

The IVs were selected and harmonized in accordance with the above criteria and outliers were excluded using the MR-PRESSO analysis. To conclude, we included a total of 365 SNPs for asthma (21 for feeling tense, 36 for feeling worry, 6 for feeling lonely, 18 for worry too long after an embarrassing experience, 23 for feeling hurt, 38 for experiencing mood swings, 34 for feeling nervous, 86 for neurociticism, 30 for feeling miserable, 36 for irritable mood, 13 for feeling guilty, 24 for feeling fed-up) (See Supplementary Table 1 for details), 395 SNPs for bronchiectasis (21 for feeling tense, 37 for feeling worry, 7 for feeling lonely, 21 for worry too long after an embarrassing experience, 28 for feeling hurt, 40 for experiencing mood swings, 35 for feeling nervous, 94 for neurociticism, 34 for feeling miserable, 38 for irritable mood, 13 for feeling guilty, 27 for feeling fed-up) (See Supplementary Table 2 for details), 385 SNPs for COPD (20 for feeling tense, 38 for feeling worry, 7 for feeling lonely, 19 for worry too long after an embarrassing experience, 27 for feeling hurt, 39 for experiencing mood swings, 35 for feeling nervous, 93 for neurociticism, 32 for feeling miserable, 37 for irritable mood, 13 for feeling guilty, 25 for feeling fed-up) (See Supplementary Table 3 for details), 383 SNPs for IPF (22 for feeling tense, 37 for feeling worry, 7 for feeling lonely, 19 for worry too long after an embarrassing experience, 26 for feeling hurt, 39 for experiencing mood swings, 33 for feeling nervous, 93 for neurociticism, 33 for feeling miserable, 37 for irritable mood, 11 for feeling guilty, 26 for feeling fed-up) (See Supplementary Table 4 for details), 394 SNPs for sarcoidosis (22 for feeling tense, 37 for feeling worry, 7 for feeling lonely, 21 for worry too long after an embarrassing experience, 28 for feeling hurt, 40 for experiencing mood swings, 35 for feeling nervous, 93 for neurociticism, 34 for feeling miserable, 38 for irritable mood, 12 for feeling guilty, 27 for feeling fed-up) (See Supplementary Table 5 for details), and 378 SNPs for sleep apnea syndrome (21 for feeling tense, 35 for feeling worry, 5 for feeling lonely, 19 for worry too long after an embarrassing experience, 27 for feeling hurt, 37 for experiencing mood swings, 33 for feeling nervous, 93 for neurociticism, 34 for feeling miserable, 35 for irritable mood, 12 for feeling guilty, 27 for feeling fed-up) (See Supplementary Table 6 for details) in MR analysis. All SNPs exhibited an F-statistic exceeding 10, indicating their suitability as robust instruments (see in Supplementary Tables 1‒6).

### Causal effects of negative emotions on chronic respiratory diseases

**Fig. 2:**
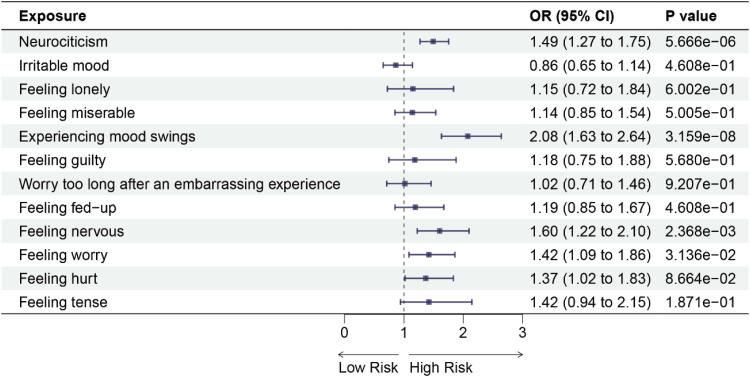
Forest plots for the causal association between negative emotions and asthma on the IVW method. IVW, Inverse Variance-Weighted; OR, Odds Ratio; 95 % CI, 95 % Confidence Interval.

**Fig. 3:**
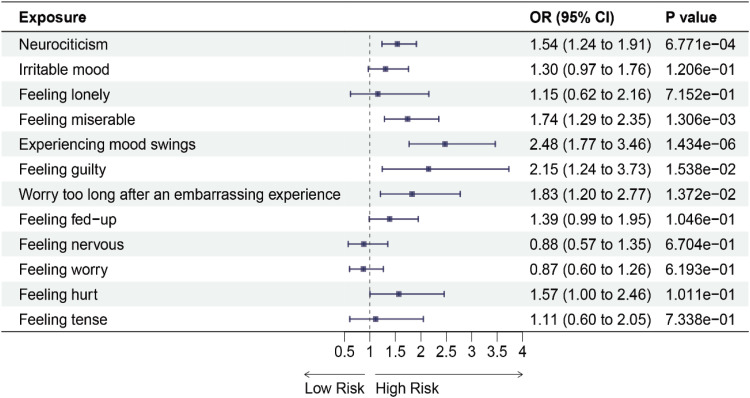
Forest plots for the causal association between negative emotions and COPD on the IVW method. COPD, Chronic Obstructive Pulmonary Disease; IVW, Inverse Variance-Weighted; OR, Odds Ratio; 95 % CI, 95 % Confidence Interval.

**Fig. 4:**
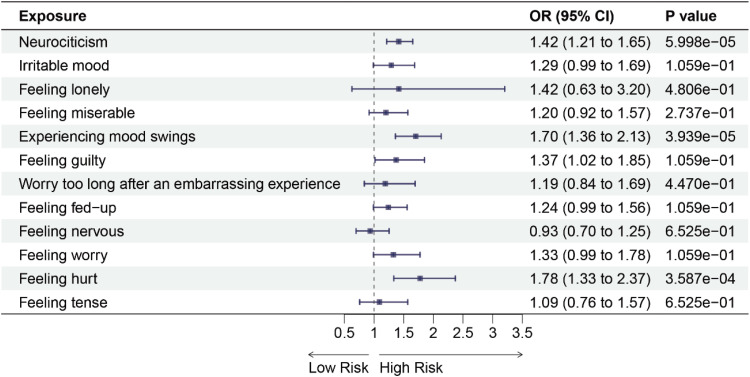
Forest plots for the causal association between negative emotions and sleep apnea syndrome on the IVW method. IVW, Inverse Variance-Weighted; OR, Odds Ratio; 95 % CI, 95 % Confidence Interval.

We initially performed a forward MR analysis to investigate the causal relationship between 12 negative emotions and 6 chronic respiratory diseases. The results revealed that using IVW analysis, neurociticism (OR = 1.49, 95 % CI: 1.27‒1.75, *p* = 5.666e-06), experiencing mood swings (OR = 2.08, 95 % CI: 1.63‒2.64, *p* = 3.159e-08), feeling nervous (OR = 1.60, 95 % CI: 1.22‒2.10, *p* = 2.368e-03) and feeling worry (OR = 1.42, 95 % CI: 1.09‒1.86, *p* = 0.0314) were associated with an increased risk of asthma (Fig. 2 and See Supplementary Table 7). Additionally, neurociticism (OR = 1.54, 95 % CI: 1.24‒1.91, *p* = 6.771e-04), feeling misery (OR =1.74, 95 % CI: 1.29‒2.35, *p* = 1.306e-03), experiencing mood swings (OR = 2.48, 95 % CI: 1.77‒3.46, *p* = 1.434e-06), feeling guilty (OR = 2.15, 95 % CI: 1.24‒3.73, *p* = 0.0154), and worry too long after an embarrassing experience (OR = 1.83, 95 % CI: 1.20‒2.77, *p* = 0.0137) were associated with an increased risk of COPD (Fig. 3 and See Supplementary Table 7). Similarly, neurociticism (OR = 1.42, 95 % CI: 1.21‒1.65, *p* = 5.998e-05), experiencing mood swings (OR = 1.70, 95 % CI: 1.36‒2.13, *p* = 3.939e-05), and feeling hurt (OR = 1.78, 95 % CI: 1.33‒2.37, *p* = 3.587e-04) were associated with an increased risk of sleep apnea syndrome (Fig. 4 and See Supplementary Table 7). No causal associations were observed between the 12 negative emotions and bronchiectasis, IPF, or sarcoidosis (Supplementary Figs. 1‒3 and See Supplementary Table 7).

Next, using the same selection criteria, we conducted a reverse MR analysis to examine the impact of asthma, COPD, and sleep apnea syndrome on the 12 negative emotional factors. The results showed no significant causal effect of asthma, COPD, or sleep apnea syndrome on negative emotional factors (*p* > 0.05, see Supplementary Table 8). Analyses based on weighted median and MR-Egger produced similar results (see Supplementary Table 8). Furthermore, the reverse MR results confirmed the robustness of the forward MR results.

### Sensitivity analysis

To further strengthen the validity of the results, a series of sensitivity analyses was conducted. Cochran's Q-statistics indicate that there is no evidence of heterogeneity among the IVs of feeling miserable (*p* = 0.285), feeling guilty (*p* = 0.053), and worrying too long after an embarrassing experience (*p* = 0.152) in this study (Supplementary Table 9), and thus, the IVW fixed-effects model was used. Further sensitivity analysis revealed that the IVW random effects model could be used for the analysis due to slight heterogeneity among neuroticism, experiencing mood swings, feeling nervous, feeling worried, and feeling hurt (*p* < 0.05, Supplementary Table 9). MR-Egger intercept analyses and MR-PRESSO analysis indicated that there was no significant horizontal pleiotropy in either direction (*p* > 0.05, Supplementary Table 9). In addition, we conducted leave-one-out analysis in order to confirm the stability of the MR results. Despite the removal of individual SNPs from the analysis, no major SNP emerged as a significant determinant that significantly altered the results (See Supplementary Table 10). Further evidence is provided by these findings that confirm the stability and integrity of the findings and confirm that negative emotions may play a causal role in chronic respiratory disease development.

### MVMR analysis of the impact of negative emotions on chronic respiratory diseases

Considering the possibility of confounding factors interfering with the results of the study, we performed Multivariate MR (MVMR) to independently assess the effect of these negative emotions on the risk of chronic respiratory diseases. In MVMR-IVW analyses adjusting for external smoking and alcohol consumption, the causal relationship between neurociticism (OR = 1.38, 95 % CI: 1.12‒1.70, *p* = 0.0028), experiencing mood swings (OR = 1.81, 95 % CI: 1.31‒2.50, *p* = 0.0003), feeling nervous (OR = 1.48, 95 % CI: 1.05‒2.09, *p* = 0.0236), feeling worry (OR = 1.47, 95 % CI: 1.10‒1.96, *p* = 0.0099) and asthma remained; the causal relationship between neurociticism (OR = 1.27, 95 % CI: 1.03‒1.60, *p* = 0.0271), experiencing mood swings (OR = 1.64, 95 % CI: 1.17‒2.29, *p* = 0.0037), feeling hurt (OR = 1.73, 95 % CI: 1.20‒2.52, *p* = 0.0037) and sleep apnea syndrome was also significant. Meanwhile, we observed that the causal associations between neuroticism, feeling miserable, feeling guilty, worrying too long after an embarrassing experience, and COPD did not persist (*p* > 0.05). However, the causal association between experiencing mood swings (OR = 1.58, 95 % CI: 1.07‒2.33, *p* = 0.0210) and COPD remained robust after adjusting for smoking and alcohol consumption. Table 2 presents the MVMR-IVW results in detail.

**Table 2:** MVMR results for causal effects after adjusting for confounding factors.

Outcomes	Exposures	Adjustment	p	OR	LowCI	UpCI
Asthma	Neurociticism	Adjusted for alcohol intake frequency	**0.0002**	1.4617	1.1933	1.7905
		Adjusted for current tobacco smoking	**0.0006**	1.3976	1.1552	1.6909
		Adjusted for both	**0.0028**	1.3766	1.1160	1.6981
	Experiencing mood swings	Adjusted for alcohol intake frequency	**0.0000**	2.0078	1.4600	2.7611
		Adjusted for current tobacco smoking	**0.0000**	1.8605	1.4138	2.4482
		Adjusted for both	**0.0003**	1.8109	1.3116	2.5005
	Feeling nervous	Adjusted for alcohol intake frequency	0.0805	1.3636	0.9631	1.9307
		Adjusted for current tobacco smoking	**0.0051**	1.5067	1.1307	2.0078
		Adjusted for both	**0.0236**	1.4844	1.0544	2.0897
	Feeling worry	Adjusted for alcohol intake frequency	**0.0405**	1.3762	1.0139	1.8679
		Adjusted for current tobacco smoking	**0.0026**	1.4761	1.1455	1.9021
		Adjusted for both	**0.0099**	1.4654	1.0962	1.9590
COPD	Neurociticism	Adjusted for alcohol intake frequency	**0.0010**	1.4944	1.1765	1.8982
		Adjusted for current tobacco smoking	0.2769	1.1403	0.8999	1.4449
		Adjusted for both	0.2795	1.1494	0.8931	1.4793
	Feeling miserable	Adjusted for alcohol intake frequency	**0.0040**	1.8311	1.2134	2.7634
		Adjusted for current tobacco smoking	0.7070	1.0935	0.6861	1.7428
		Adjusted for both	0.2406	1.3138	0.8328	2.0727
	Experiencing mood swings	Adjusted for alcohol intake frequency	**0.0000**	2.3639	1.6463	3.3943
		Adjusted for current tobacco smoking	**0.0454**	1.4934	1.0082	2.2122
		Adjusted for both	**0.0210**	1.5817	1.0714	2.3349
	Feeling guilty	Adjusted for alcohol intake frequency	**0.0209**	1.8599	1.0985	3.1492
		Adjusted for current tobacco smoking	0.9832	0.9931	0.5205	1.8948
		Adjusted for both	0.9119	1.0308	0.6025	1.7636
	Worry too long after an embarrassing experience	Adjusted for alcohol intake frequency	0.3637	1.2467	0.7747	2.0063
		Adjusted for current tobacco smoking	0.6582	1.1307	0.6562	1.9483
		Adjusted for both	0.4926	1.1909	0.7229	1.9620
Sleep apnea syndrome	Neurociticism	Adjusted for alcohol intake frequency	**0.0228**	1.2618	1.0329	1.5414
		Adjusted for current tobacco smoking	**0.0001**	1.4167	1.1844	1.6946
		Adjusted for both	**0.0271**	1.2740	1.0278	1.5791
	Experiencing mood swings	Adjusted for alcohol intake frequency	**0.0156**	1.4962	1.0794	2.0739
		Adjusted for current tobacco smoking	**0.0004**	1.6850	1.2655	2.2435
		Adjusted for both	**0.0037**	1.6401	1.1739	2.2913
	Feeling hurt	Adjusted for alcohol intake frequency	**0.0072**	1.6787	1.1505	2.4494
		Adjusted for current tobacco smoking	**0.0027**	1.6615	1.1928	2.3144
		Adjusted for both	**0.0037**	1.7341	1.1953	2.5159

COPD, Chronic Obstructive Pulmonary Disease; MVMR, Multivariable Mendelian Randomization; OR, Odds Ratio; CI, Confidence Interval. Font-weight indicates that the relationship has statistical significance.

## Discussion

In this study, we employed both two-sample MR and MVMR analyses to explore the causal relationships between 12 negative emotional factors (feeling tense, feeling worry, feeling lonely, worry too long after an embarrassing experience, feeling hurt, experiencing mood swings, feeling nervous, neurociticism, feeling miserable, irritable mood, feeling guilty, and feeling fed-up) and 6 chronic respiratory diseases (asthma, COPD, bronchiectasis, sarcoidosis, sleep apnea syndrome, and IPF). The study concluded that neuroticism and experiencing mood swings are associated with an increased risk of three chronic respiratory diseases (asthma, COPD, and sleep apnea syndrome). Additionally, feeling nervous and feeling worry are associated with an increased risk of asthma; misery, feeling guilty, and worrying too long after an embarrassing experience are associated with an increased risk of COPD; feeling hurt is associated with an increased risk of sleep apnea syndrome. No causal relationship was found between the negative emotions and bronchiectasis, sarcoidosis, or IPF. Sensitivity analyses further supported these conclusions, emphasizing the reliability and stability of the findings. Finally, MVMR analyses showed that negative emotions are associated with asthma and sleep apnea syndrome independently of potential confounding variables. For COPD, only the causal effect of experiencing mood swings was observed to persist.

The present study identified neurociticism and experiencing mood swings as the most common risk factors for chronic respiratory diseases. Neuroticism is a stable and heritable higher-order personality trait, defined as the tendency to experience negative emotions. Neuroticism is an important factor in most personality assessments and models, and has been found to be associated with various health outcomes and increased mortality risk. MR results have shown that neuroticism is significantly associated with an increased risk of stroke and a higher frailty index.^18^ A meta-analysis suggested that lower neuroticism is associated with a reduced risk of arthritis.^19^ Neuroticism is also closely related to the occurrence of cardiovascular diseases. Neuroticism personality traits are linked to smaller ventricles, poorer function, lower left ventricular mass, higher myocardial fibrosis, and increased arterial stiffness.^20^ An MR study found extensive polygenic overlap (20 %‒97 %) between neuroticism and cardiovascular disease, suggesting that genetics may mediate the impact of neuroticism on cardiovascular health.^21^ In the lungs, higher neuroticism is associated with lower peak expiratory flow and a higher risk of dyspnea.^22^ Experiencing mood swings, defined as frequent, sudden, and unpredictable changes in emotional state, is a fundamental personality trait that can have both positive and destructive effects. Severe mood swings can be classified as psychiatric disorders, such as bipolar disorder. Previous studies have shown that experiencing mood swings is associated with an increased incidence of cardiovascular and cerebrovascular diseases, as well as certain gynecological conditions.^23^ Additionally, genetically determined mood swings increase the risk of endometriosis and adenomyosis.^24^ Mood swings are also linked to chronic inflammatory diseases, such as psoriasis.^25^ These findings are consistent with the present results and warrant further investigation into the underlying mechanisms.

Furthermore, we observed that different chronic respiratory diseases have unique emotional risk factors. Feeling nervous and feeling worried are associated with an increased risk of asthma; feeling misery, feeling guilty, and worrying too long after an embarrassing experience are associated with an increased risk of COPD; feeling hurt is associated with an increased risk of sleep apnea syndrome. Currently, no literature explores the specific associations between these negative emotions and various chronic respiratory diseases. However, psychological factors such as negative emotions have been shown to affect the progression and treatment of chronic respiratory diseases. Subjective loneliness affects treatment outcomes in COPD patients undergoing pulmonary rehabilitation.^26^ In animal studies, different types of stress (both acute and chronic) are associated with exacerbation of allergic inflammation markers.^27^ In asthma patients, daily variations in negative emotions are positively correlated with changes in the fraction of nitric oxide in exhaled breath.^28^ No associations were observed between negative emotions and bronchiectasis, sarcoidosis, or IPF, highlighting the need for more prospective studies to explore these relationships.

There is a complex interaction between emotions and chronic respiratory diseases, and the underlying pathophysiological mechanisms remain incompletely understood. On one hand, in chronic respiratory diseases, a marked increase in emotional responses such as depression and anxiety has been observed.^29^ Moreover, chronic respiratory patients with comorbid anxiety, depression, and other conditions tend to have more severe symptoms and a worse prognosis. Li et al^30^ found that in asthmatic mice, psychosocial stress can activate the hypothalamic-pituitary-adrenal axis, impair glucocorticoid sensitivity and function, and increase airway hyperresponsiveness and inflammation, leading to asthma exacerbation. Functional magnetic resonance imaging studies by Ritz et al. also suggest that, in asthma patients, the degree of airway constriction induced by negative emotional stimuli is associated with stronger responses to these stimuli in the dorsolateral prefrontal cortex and midcingulate cortex.^31^ In asthma patients with more severe airway inflammation and poorer asthma control, there is a reduction in activation of multiple cortical and subcortical regions related to emotional processing and respiratory control. Herigstad and his colleagues have offered deeper insights into the interaction between brain activity related to respiratory symptoms and behavior, physiology, and psychology. They suggest that negative emotions and attention can serve as modulatory factors within the nervous system, adjusting the prior or weight of incoming sensory information, thereby influencing symptoms.^32^ They observed activation of the medial prefrontal cortex and anterior cingulate cortex in COPD patients. Compared to the high symptom load group, the low symptom load group (with lower anxiety and general symptom burden) exhibited significantly enhanced brain activity in the anterior insula.^33^ The anterior insula is involved in processing body sensations and perception-related functions.

On the other hand, emotions can also influence chronic respiratory diseases, involving genetic and hormonal levels. Kawakami et al^34^ found that in asthma exacerbation triggered by emotional stress, functional SNPs in the stress-related μ-opioid receptor gene (OPRM1; A118G SNP, rs1799971) may induce eosinophilic inflammation in mouse lungs by increasing the generation of effector Th2 cells in peripheral lymph nodes. Pincus-Knackstedt et al^35^ demonstrated that prenatal stress increases the susceptibility of adult offspring mice to airway inflammation, as evidenced by increased anxiety levels in the adult offspring, reduced expression of corticotropin-releasing hormone in the paraventricular nucleus, and the ability of antigen-presenting cells from prenatally stressed offspring to trigger clonal expansion of Th2 cells in vitro. The complex role of emotions in chronic respiratory diseases suggests that actively managing emotional responses could be an important strategy for disease prevention and improving prognosis. However, more basic and prospective clinical research is needed to explore the underlying mechanisms.

Less direct associations include the possibility that negative emotions may promote disease through various mechanisms, such as accelerating aging, fostering substance addiction, altering lifestyle, and modifying the microbiome. Neuroticism is significantly associated with dependence on drugs, alcohol, and tobacco, which are risk factors for many diseases.^36^ Neuroticism may also promote disease by influencing age-related changes in immune phenotypes. Additionally, individuals with low neuroticism exhibit higher activity diversity, including volunteering, sports, hobbies, and learning, which may serve as protective factors against diseases. Mood swings may influence redox balance, endocrine function, inflammation, and immunity.^37^ Increased serum inflammatory biomarkers, including COX-2, arachidonic acid, IL-6, and tumor necrosis factor-alpha, have been observed in patients with bipolar disorder.^38^ Neuroticism may drive neuropsychological and gut microbiota characteristics that promote obesity.^39^ Potential links between bipolar disorder and gut microbiota have also been suggested.^40^ However, these associations are insufficient to elucidate the causal relationship and underlying mechanisms between negative emotions and disease. In fact, in addition to negative emotions, positive emotions also contribute to disease progression, often serving as a protective factor. Both are balanced through complex psychological and physiological mechanisms. Studies on negative emotions and disease must consider the interaction between positive and negative emotions and their combined effects on health.

However, this study has limitations that necessitate cautious interpretation. Firstly, the GWAS summary data that we used were derived exclusively from European populations. This emphasizes the need for caution when generalizing the findings to other ethnic groups with different lifestyles and cultural backgrounds. Secondly, the assessment of negative emotions was conducted using the Eysenck Personality Questionnaire Revised Short Form, which does not include the evaluation of emotion duration and intensity. And the completion of the questionnaire may be influenced by subjective factors, potentially leading to bias in the results. Furthermore, due to limitations in the GWAS summary data, we could not obtain clinical data related to the population, preventing subgroup analysis and disease severity analysis. Finally, considering the inherent challenges of MR analysis, which relies on the random allocation of genetic variants, it cannot completely eliminate the influence of pleiotropy. There is a reasonable possibility that genetic variants within the genome may simultaneously affect multiple phenotypes. Additionally, interactions between multiple exposures may lead to collinearity, confounding, interactions, and estimation instability, thereby affecting the causal effect estimates. Therefore, further research is needed to investigate the underlying mechanisms of these associations.

## Conclusion

Utilizing extensive GWAS summary data, this study provides robust evidence supporting the causal relationships between negative emotions (including neurociticism, experiencing mood swings, feeling nervous, feeling worried, and feeling hurt) and an risk of chronic respiratory diseases, such as asthma, COPD, and sleep apnea syndrome. We did not find causal relationships between negative emotions and bronchiectasis, sarcoidosis, or IPF. These findings underscore the importance of mental health in the prevention and treatment of chronic respiratory diseases and offer new insights into the genetic aspects of the connection between negative emotions and these conditions. However, larger-scale prospective studies and in-depth mechanistic research are necessary to further validate and fully elucidate the causal relationships between negative emotions and various chronic respiratory diseases.

## Funding

The authors received no specific funding for this work.

## Ethics approval and consent to participate

The GWAS statistics used in this study are publicly available. All original studies providing these data had received ethical approval from their respective Institutional Review Boards and obtained informed consent from participants. As the data are de-identified and aggregated, no additional ethical approval was required.

## Data availability

All relevant data are within the paper and its Supporting Information files.

## CRediT authorship contribution statement

**Hui Chen:** Writing – original draft, Writing – review & editing, Visualization, Software, Formal analysis, Conceptualization. **Jinping Zhu:** Writing – review & editing, Methodology, Visualization, Data curation, Conceptualization. **Zewen Yang:** Writing – review & editing, Methodology, Conceptualization. **Yimin Zha:** Software, Methodology, Conceptualization. **Junjie Yang:** Methodology, Conceptualization.

## Declaration of competing interest

The authors declare no conflicts of interest.

## Appendix Supplementary materials

Image, application 1

Image, application 2
