# Supplementary material for: The causal role of multiple negative emotions in chronic respiratory diseases: A two-sample Mendelian randomized study
Source: Clinics (Sao Paulo). 2025 Nov 7;80:100819. doi: 10.1016/j.clinsp.2025.100819 (PMC12639550; doi:10.1016/j.clinsp.2025.100819)

**CLINICS-D-25-00006_Supplementary Material_Figures**

**Supplementary Figures 1** Forest plots for the causal association between negative emotions and bronchiectasis on the IVW method. IVW, Inverse Variance-Weighted; OR, Odds Ratio; 95% CI, 95% Confidence Interval.


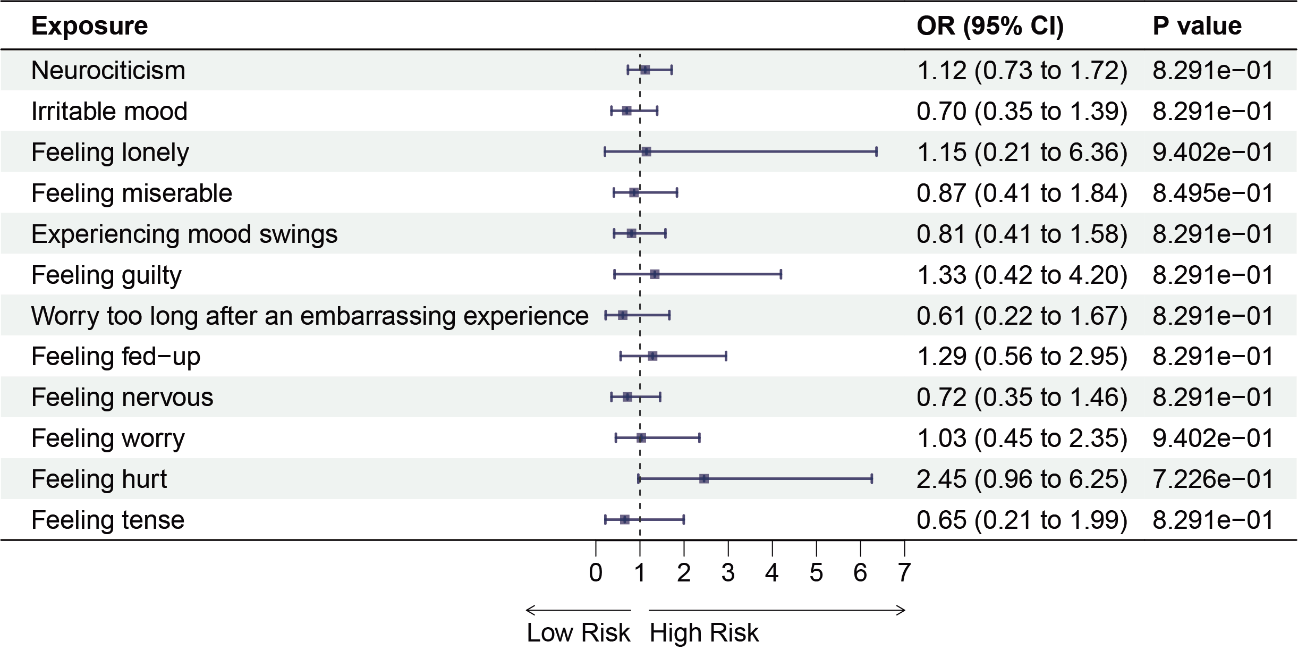


**Supplementary Figures 2** Forest plots for the causal association between negative emotions and IPF on the IVW method. IPF, Idiopathic Pulmonary Fibrosis; IVW, Inverse Variance-Weighted; OR, Odds Ratio; 95% CI, 95% Confidence Interval.


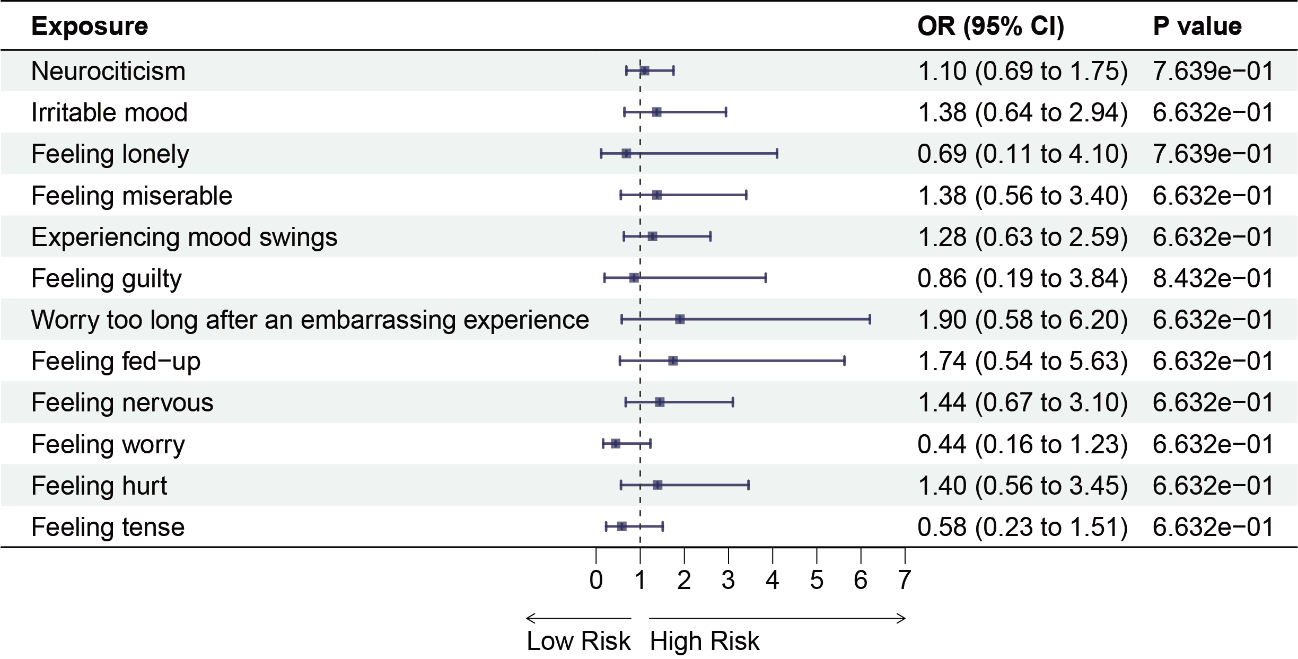


**Supplementary Figures 3** Forest plots for the causal association between negative emotions and sarcoidosis on the IVW method. IVW, Inverse Variance-Weighted; OR, Odds Ratio; 95% CI, 95% Confidence Interval.


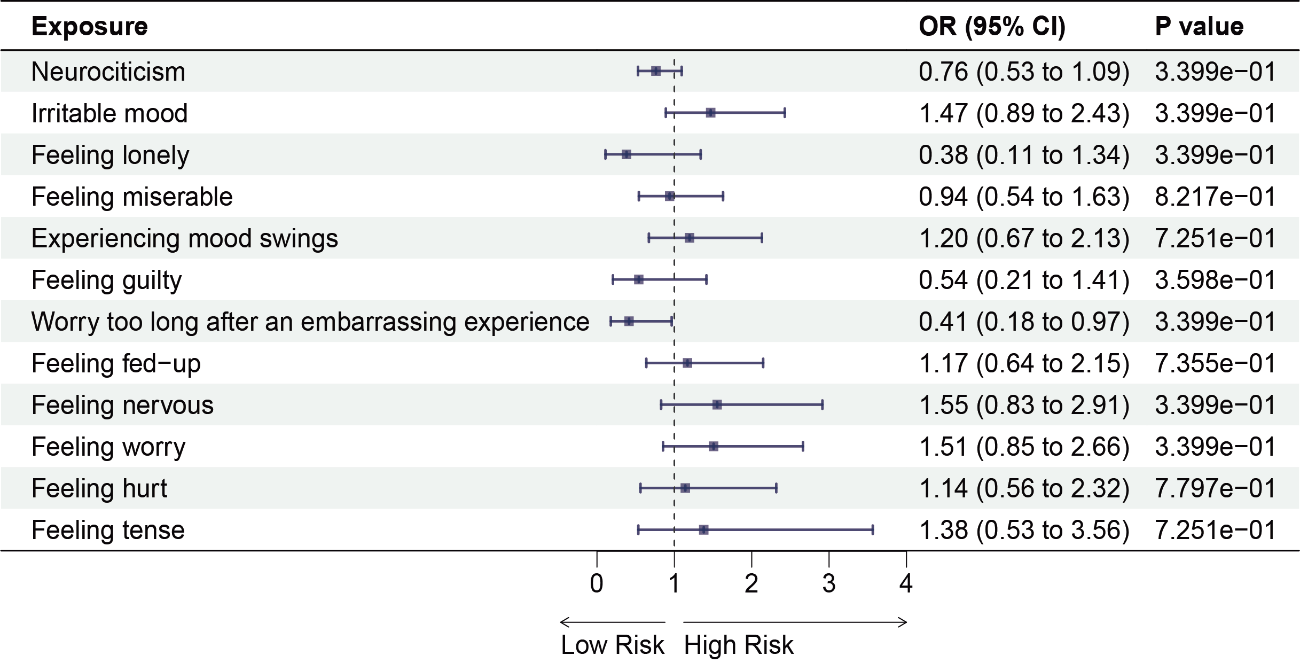

Supplement: Supplementary file 1 [file mmc1.docx]
